# Poor Sleep Moderates the Association Between Generalized Anxiety and Hippocampal Volume Among Older Adults

**DOI:** 10.1093/geroni/igab046.3295

**Published:** 2021-12-17

**Authors:** Desiree Bygrave, Regina Wright

**Affiliations:** 1 Benedict College, Benedict College, South Carolina, United States; 2 University of Delaware, University of Deleware, Delaware, United States

## Abstract

Poor sleep is common among older adults, and associated with hippocampal atrophy -- a strong predictor of memory decrements. Underlying this association are psychosocial risk factors, such as generalized anxiety, that may further exacerbate poor sleep and brain pathology. Given that poor sleep and generalized anxiety are often comorbid, there is a critical need to establish whether generalized anxiety is related to hippocampal volume among poor sleepers. To address this gap, this cross-sectional study examined the relationship between generalized anxiety (GAD-7), and total hippocampal volume, and whether it varied as a function of sleep quality (PSQI Total < 5 good sleepers; PSQI Total ≥ 5 poor sleepers). Data were analyzed from 165 older adults (mean age = 68.48y, 33% male, 41% African American), free of major disease. Linear regression analysis, adjusting for sex, race, education and depression, showed a statistically significant Generalized Anxiety x Sleep interaction for hippocampal volume (p=.02). Further probing of this interaction revealed that among poor sleepers, greater generalized anxiety was associated with lesser hippocampal volume (p=.01). Findings suggest generalized anxiety may influence hippocampal volume in the context of poor sleep among older adults. As poor sleep is associated with age-related neurodegeneration, our findings suggest that improvements in sleep quality may reduce the impact of generalized anxiety on hippocampal volume in older adulthood. Future research should examine whether generalized anxiety mediates relations of sleep quality to specific memory outcomes.

